# The impact of bilinguality and language context on the understanding of epistemic adverbs in health communication: the case of English and Russian

**DOI:** 10.3389/fpsyg.2023.1179341

**Published:** 2023-06-16

**Authors:** Vanda Nissen, Renata F. I. Meuter

**Affiliations:** School of Psychology and Counselling, Faculty of Health, Queensland University of Technology (QUT), Brisbane, QLD, Australia

**Keywords:** uncertainty in health communication, epistemic adverbs, bilingual patients, cultural consensus analysis, multidimensional scaling, semantic space

## Abstract

**Aim:**

To explore how English epistemic adverbs, as used in health communication, are understood by speakers depending on their first language (L1) and language context.

**Methods:**

We used an online dissimilarity rating task with paired doctors' opinions which differed only with respect to the embedded epistemic adverbs (e.g., This treatment *definitely* has side effects vs. This treatment *possibly* has side effects). In order to evaluate the possible effect of one's L1, we compared the ratings of English-speaking monolinguals and Russian-English bilinguals in Australia (Study 1). To evaluate the impact of language context, we compared the ratings of Russian-English bilinguals in Australia and Russia (Study 2). The data were interpreted using classical multidimensional scaling (C-MDS) analysis, complemented by cultural consensus analysis and hierarchical cluster analysis.

**Results:**

The C-MDS analyses returned statistically acceptable results. Intragroup consensus was evident for all speaker groups. They all clustered the high confidence adverbs (*clearly, definitely, and obviously*) and the hearsay adverbs (*presumably and supposedly*) similarly. Effects of L1 were seen: for example, unlike the monolinguals, the Russian bilinguals did not include *evidently* with the high confidence adverbs (Study 1). An effect of context was also evident: Russian-English bilinguals in Australia most resembled the monolinguals in their understanding of epistemic adverbs. The way Russian-based bilinguals clustered epistemic adverbs reflected a less nuanced understanding (Study 2).

**Conclusion:**

The subtle differences in how adverbs of likelihood and doubt are understood in health communication suggest extra care is needed when conveying risk and uncertainty to patients from diverse linguistic and/or cultural backgrounds to ensure mutual understanding and mitigate against miscommunication. The impact of L1 and language context on one's understanding highlights the need to explore more widely how epistemic adverbs are understood by diverse populations and, in doing so, improve healthcare communication practices.

## Background

Imagine you are a patient for whom English is the second language, accessing your healthcare in a monolingual English setting. If your doctor says “*You are* ***probably*** *going home tomorrow,”* how certain are they that you will be discharged the next day compared to if they said, “*You are* ***possibly*** *going home tomorrow”*? Not only may you interpret these statements differently, but you may also interpret your doctor's statement as having a higher likelihood of occurrence than they intended to communicate. While the resulting miscommunication may be distressing to you, thankfully, it is unlikely to have significant negative implications on your health.

Unfortunately, miscommunication in healthcare is common: it exacerbates uncertainty (e.g., regarding diagnosis) and negatively impacts patient outcomes because patients may get less attention, which may result in misdiagnoses and incorrect treatment (Hickman et al., 2009; Bailey and Arciuli, 2020). With bilingual patients, uncertainty is further increased through language barriers, challenges with comprehension, cultural estrangement, and other social and psychological factors (Andrulis and Brach, 2007; Butow et al., 2011; White et al., 2018; Alqurashi, 2019; Patriksson et al., 2019a,b; Rashoka et al., 2022). When communicating risk and uncertainty, some words, such as epistemic adverbs (e.g., *likely, possibly, and probably*), are particularly critical to ensure a proper understanding of health information because they indicate the likelihood of an event or condition. Along with other means of epistemic modality, they constitute a significant part of health communication, especially where they form part of medical advice and recommendations, and of doctor and patient opinions (Heritage and McArthur, 2019).

As research reveals, even monolingual speakers of the same language (in this case, English) may comprehend epistemic adverbs slightly differently, such as when they are from different speech communities (e.g., Australia and Montreal in Canada) (Segalowitz et al., 2016). In Segalowitz et al.'s (2016) study, 12 epistemic adverbs (*apparently, certainly, clearly, definitely, evidently, likely, obviously, probably, possibly, presumably, reportedly*, and *supposedly*) were used as stimuli. The adverbs were embedded in pairs of sentences modeling a first doctor's opinion and a second doctor's opinion, each pair differing only in terms of the embedded epistemic adverb. There were 98 sentence pairs, each accompanied by a 9-point rating scale. In Study 1, Australian English speakers (n = 69) and Canadian English speakers (n = 19) performed a dissimilarity rating task with the sentence pairs. The data were submitted to exploratory multidimensional scaling (MDS) and analyzed using a combination of cultural consensus analysis, weighted-data classical-MDS (C-MDS), and cluster analysis.

Each group of speakers differentiated a similar set of adverbs reflecting “confidence” from the others, however, there were some differences in clustering. First, Canadian English speakers clustered the adverb *obviously* together with other confident adverbs (*definitely, clearly, and certainly*), while speakers of Australian English did not. Second, in the Australian sample, *evidently* appeared in a cluster along with *obviously*, whereas the Canadian sample clustered it with *probably*. Third, while both Australian and Canadian participants grouped together adverbs of indirect knowledge (*presumably, apparently, and supposedly*), the fourth adverb in this cluster was different: *possibly* in the Canadian sample and r*eportedly* in the Australian sample. These cluster patterns highlight how uncertainty adverbs may be understood differently even by speakers of the same language. A possible explanation is that the Canadian participants, residents of Montreal, were exposed to the French language on a daily basis (the effect of language context).

This finding that one's language environment and experience can impact the representation of meaning suggests that there is value in extending our knowledge of how uncertainty is understood to more diverse speaker populations. Accordingly, the current research builds on Segalowitz et al.'s (2016) and Meuter et al.'s (2018) findings and, in two studies, investigates how adverbs of certainty and uncertainty are understood by Russian speakers of English compared to monolingual Australian English speakers, thus exploring the impact of bilinguality (i.e., the impact of Russian as the first language (L1)) (Study 1), and whether this understanding differs between Russian-English bilinguals^1^ residing in Australia vs. Russia, thus tracking the impact of language context (Study 2).

Speakers of other languages in monolingual Australia all form separate groups of language users, unlike Montreal, where English and French are widely spoken and co-occur in the environment (e.g., advertising, education, and healthcare). We chose Russian-English bilinguals as our speaker group because of the existing cultural and linguistic differences. In Australia, the Russian speaking community counts more than 50,000 people from the former USSR (ABS, 2021), and individuals from this group often have a different attitude toward and expectations of medicine and healthcare in Western countries compared to their home country (Grabbe, 2000; Shpilko, 2006; Team et al., 2013; Phung et al., 2020). They are more accustomed to a paternalistic approach where health practitioners (HPs) take full responsibility for a patient's condition (Shulyaev et al., 2020). Consequently, Russian patients tend to adopt a passive role in health communication. Research also shows poor health literacy among Russian and Ukrainian speaking immigrants, especially in the 50+ age group (Team et al., 2013). Conversations about one's health may involve emotional expression, and here Russian patients are also likely to differ from Western patients. Russian patients often express their emotions physically rather than verbally (Zisberg, 2017).

In addition to cultural differences, there are linguistic differences between English and Russian, including in the understanding of epistemology. Epistemic adverbs convey the speaker's stance in relation to the information presented, and they are common in English. Wierzbicka (2006) explained the large repertoire of epistemic adverbs in English by the motivation of the modern English speaker not to impose their views on the addressee. In contrast, Russian epistemic modality is centered on the speaker's point of view, which is transmitted to the addressee. Russian also employs epistemic particles and other discourse markers to mark likelihood and uncertainty, while English is comparatively poor in this respect (Wierzbicka, 2006). For example, in Russian, the closest equivalent to the adverb *reportedly* is a parenthetical expression по имеющимся сведениям *(po imejushchimsya svedeniyam)*, which can be translated as “according to the existing information.” This translation is only partially equivalent to *reportedly*. Although Russian has epistemic adverbs, some semantic nuances may still exist. For example, the Russian epistemic adverb ясно *(jasno)*, depending on the context, can be translated as *clearly, evidently*, and *obviously*.

To understand how meanings may be represented in bilingual and multilingual speakers, and account for semantic nuances that occur in one but not the other language, there are models of lexical representation that provide useful frameworks. For example, according to the Distributed Conceptual Feature Model, the layer of word meanings contains conceptual representations that are shared between two languages, and elements that are language specific. Concrete words are more often represented as sharing a conceptual representation between the two languages, whereas abstract words are more often represented as having language-specific conceptual representations (de Groot, 1997). Abstract words may be more dependent on context for their interpretation, and they are more likely to differ across languages because they share fewer conceptual features compared to concrete words. Therefore, abstract words may possess only roughly similar equivalents in another language (Kroll and Stewart, 1994). In second language (L2) vocabulary, they are more difficult to learn (de Groot and Keijzer, 2000) and they are retained worse than concrete words (Altarriba and Basnight-Brown, 2012).

Because epistemic adverbs are abstract and given Segalowitz et al.'s (2016) findings, Russian-English bilinguals may be expected to understand English epistemic adverbs somewhat differently from Australian English monolinguals because of their knowledge and use of the Russian language, even when living in the same cultural context (i.e., English-speaking Australia). Furthermore, different groups of Russian-English bilinguals may not share the same understanding of the English adverbs of (un)certainty because they do not share the same language context. For example, while a Russian-English speaker in Australia typically would be immersed in an English-speaking environment, the same is not true for a Russian-English speaker in Russia who also has far fewer occasions to use the English language.

To explore the impact of bilinguality and of language context on how English epistemic adverbs are understood, we carried out two studies, each focused on the understanding of these adverbs in a health context. Study 1 focused on Russian-English bilinguals and English-speaking monolinguals in Australia to establish how similar (or different) their understanding of epistemic adverbs is as a function of bilinguality. Russian and English differ markedly in how uncertainty and likelihood are expressed. Does that different use and understanding of epistemological expressions in Russian speakers as a first language impact how they understand epistemic adverbs in English? The answer to this question is important for health communication, given that clarity regarding one's health condition and suggested treatments rely on the patient and HP understanding the degree of likelihood and uncertainty expressed by the other.

Study 2 focused on a comparison of Russian-English bilinguals based in Australia and their counterparts resident in Russia, thus enabling an evaluation of the impact of the language context on the way these epistemic adverbs are represented semantically. Both speaker groups are native Russian speakers, but only the bilinguals who are residing in Australia not only use English on a daily basis but have been immersed in the English language and therefore have familiarity with the use of English epistemic adverbs in the Australian host language context. Does that experience subtly impact how the meanings of English epistemic adverbs are represented?

Following Segalowitz et al.'s (2016) approach, rather than asking our speakers outright how similar or different in meaning the English epistemic adverbs were, we embedded the adverbs in sentences, where each sentence represented a doctor's opinion (e.g., Your leg is *probably* broken), thus unambiguously specifying a health context within which the epistemic adverb is interpreted to signal the doctor's degree of certainty. The use of contextualized sentences ensures that the interpretation of the sentences, and by extension therefore also the embedded adverbs, is confined to a unique context, in this case, healthcare. The sentences were paired, differing only with respect to the embedded adverb. The task was to rate how dissimilar in meaning the sentence pairs were (e.g., Your leg is *probably* broken vs. Your leg is *possibly* broken). The resulting data (consisting of dissimilarity ratings specific to adverb pairings) was then subjected to Multi-Dimensional Scaling (MDS), similar to Segalowitz et al. (2016). Importantly, we considered the extent to which each speaker group agreed on the meaning of the epistemic adverbs, recognizing not only that there may be variation within a group but also that any such variation may well be different from that observed in another speaker group (e.g., contrasting monolingual speakers of English and speakers of English as a second language). To incorporate the within-group consensus component, we used cultural consensus analysis.

In Study 1, we contrasted the data collected from monolingual speakers of Australian English with that obtained from Australian-based Russian-English bilinguals and explored whether the Russian-English bilinguals showed similar understandings of the English epistemic adverbs. Because the data collected from the Australian monolinguals constituted a partial replication of Segalowitz et al. (2016) 6 years after their data was collected, we also compared the patterns we obtained with those obtained by them in their Study 1. In Study 2, we contrasted data from a Russian-English bilingual speaker group based in Russia (Russian bilinguals) with that obtained from the Australian-based Russian-English bilinguals (Australian bilinguals) to explore the degree of consensus within these bilingual speaker groups regarding the semantic representation of the epistemic adverbs and the extent to which the language context impacted on the semantic representations in each group. The data were analyzed with C-MDS, supplemented by cultural consensus analysis and hierarchical cluster analysis.

## Method - Studies 1 and 2

This section describes the method and materials, noting that these are the same for both studies.

### Participants

A total of 168 participants were recruited from various sources, including the Russian community in Australia, sports organizations, professional associations, universities in Australia and Russia, personal contacts, and churches. To be eligible for participation, individuals had to be at least 18 years old and be native, L1 speakers of Russian or Australian English. We excluded participants who failed the “honesty” question^2^ in the survey (n = 36), resulting in 132 completed surveys. A few participants (n = 5) did not meet the L1 criteria for inclusion and were therefore excluded from the data. After these exclusions, we had the following numbers of participants: Australian monolinguals (n = 77); Russian-English bilinguals (n = 50). We further excluded two bilinguals because they did not meet the residency criteria (i.e., they resided somewhere other than Australia or Russia). We retained one participant living in Ukraine based on the demographic information provided (resident in a Russian speaking region of Ukraine, L1 Russian, L2 English). (See Table 1 for details of the bilinguals' place of birth and level of education.) The remaining 48 Russian-English bilinguals formed two groups based on their place of residence: Australian bilinguals (n = 26) and Russian bilinguals (n = 22).

**Table 1:** Place of birth and the highest level of education of the Russian-English bilinguals based on residency (Australia vs. Russia).

**Russian-English bilinguals**		
**(*****n*** = **38)**		
**Place of residence**	**Australia (*****n*** = **21)**	**Russia (*****n*** = **17)**
**Place of birth**		
Russia	12	15
Ukraine	4	2
Belarus	2	
Azerbaijan	1	
Kazakhstan	1	
Tajikistan	1	
**Education**		
High school		1
Undergraduate	10	6
Postgraduate	11	10

The number of individuals (n) is presented in brackets.

Further exclusions applied are as follows. For our bilingual groups, we excluded participants reporting weak speaking and listening ability in English (rated ability <3 on a 5-point Likert scale, from 1 = *no ability at all* to 5 = *fluent*). From the Australian bilinguals, we further excluded participants whose daily use of English was reported to be lower than 50%, because our purpose was to establish whether Australian bilinguals, who were immersed in the Australian English context, differed in their understanding from the Russian bilinguals (Study 2). Additionally, we asked our bilingual participants to rate how well they spoke and understood English focusing on their use and understanding of English when communicating. A 5-point Likert scale was used, ranging from “*I cannot express/understand others very much at all in the language*” to “*I can express myself/I can understand native speakers in all or almost contexts, using all or nearly all expressions that native speakers typically use*.” Of the bilinguals retained for analysis, more than half of the Australian bilinguals could express themselves in a native-like way in all or most contexts (52%), with the remainder able to express themselves on unfamiliar topics but without necessarily knowing all the right terminology (48%). In addition, the majority were able to understand native English in almost all contexts, including the expressions used (62%), with the remainder able to do so in most contexts even when not all expressions were entirely understood (38%). In contrast, most of the Russian bilinguals (76%) were able to express themselves on unfamiliar topics, without necessarily knowing all the right terminology. A similar pattern was reflected in their understanding of native English, where most Russian bilinguals (65%) were able to do so in most contexts even when not all expressions were entirely understood. In other words, overall, the Russian bilinguals were less proficient in their use and understanding of English, consistent with living and working in a Russian language context.

The number of Australian bilinguals retained for analysis was 21 (M_age_ = 38, age range = [23–58]; all women; median daily use of English = 70%; range = [50–95%]). For the Russian bilinguals retained for analysis (n = 17; M_age_ = 42, age range = [20–64]; 13 women), the reported median daily use of English was 30% (range = [1–60%]). For the bilinguals we also recorded knowledge of other languages, recognizing that bilinguals often speak more than two languages (Calafato, 2020). On average, 12% of bilinguals spoke other languages in addition to English and Russian. We note that all bilinguals retained for analysis reported that their strongest language was Russian. They acquired English formally in school, in Russia, from the age of 10 (c.f. Ustinova, 2005). Thus, the Australian bilinguals had a formal basis in English prior to arriving in Australia.

For our monolinguals, to enable a clear comparison of our monolingual ratings with those reported by Segalowitz et al. (2016), we further excluded participants who reported any knowledge of another language, did not speak English most of the time, and were older than 55 years. These further exclusion criteria resulted in 62 monolinguals for analysis (M_age_ = 22.77, age range = [18–50]; 51 women).

On completion of the survey, participants were invited to participate in a prize draw of three $50 Amazon gift cards. The choice of the prize draw was based on the evidence that prize draws seem more effective than prepaid incentives in improving response rates (Bosnjak and Tuten, 2003). Participants who were first-year Psychology students at the institution where this study was conducted could elect to receive course credit instead.

### Stimuli

The target words used were the following 12 epistemic adverbs: *apparently, certainly, clearly, definitely, evidently, likely, obviously, probably, possibly, presumably, reportedly*, and *supposedly*. They were combined in 66 different pairs and embedded in carrier sentences that reflected a first vs. a second medical opinion (e.g., *This could certainly cause some cramps* vs. *This could supposedly cause some cramps*). Each adverb occurred in 11 different carrier sentences and only once in each. Furthermore, each adverb occurred five or six times out of the total 11 occurrences in the First and Second opinions. Dissimilarity ratings for each sentence pair were obtained using a 9-point Likert-type dissimilarity rating scale (ranging from 1 = *not at all different* to 9 = *extremely different*). Warm up and filler trials were formed using eight additional expressions of confidence and doubt, ones that were similar in intention to the target sentences and enabled the formation of similar pairs of sentences. One such example is the following sentence pair: *From reports I've seen, this will require chemotherapy* vs. *I'm positive that this will require chemotherapy*.

The sentence pairs were organized into 98 trials, consisting of the 66 target adverb comparisons interspersed by 28 filler pairs, and four warm-up trials. In the survey, 33 different carrier sentences were used, each associated with one filler and two adverb expressions. Sentence pairs were quasi-randomized so that no carrier sentence and no adverb occurred in consecutive trials. Eight more sentence pairs were used in the survey instructions, six of which contained filler expressions and two contained adverbs. To ensure that participants took regular pauses throughout the survey, three equally spaced breaks were introduced. These breaks were active: each contained three anagrams to be solved. Solutions were provided. Responses to practice questions, filler pairs, warm-ups, and anagrams were excluded from the subsequent analysis.

### Language background questionnaire (LBQ)

The Language Background Questionnaire captured basic information about participants (e.g., age, gender, country of birth, and country of residence), as well as details of their language background (e.g., self-reported proficiency in speaking, listening, reading, and writing in each language), employment status, and the highest level of educational attainment.

### Final survey procedure

The survey was created using Qualtrics. Participants answered the questionnaire online from home or another location of their choice. Completion of the survey constituted informed consent. The survey was online, and the survey link was distributed by email. It remained open for 11 weeks.

We recorded IP addresses to keep track of unique visitors to the survey (n = 452), however, we did not put any IP restrictions in place bearing in mind that there could be several members of one household participating in our survey. There were no duplicate entries from the same IP address among the completed surveys. The participation rate was 78% (calculated as a percentage of total unique visitors, with 360 participants engaging in the survey). Of those who engaged in the survey, 168 participants completed the survey (46% completion rate). The mean completion time of the survey was 102 min (range = [10–1,668 min]). The limit we set for the survey to be inactive before it was marked as recorded was 24 h. However, the participants may have logged in before the 24-h period was up (thus resetting the inactivity marker), and it was extended to 72 h. This explains why the completion time was longer for some participants.

### Data collection and analysis

The survey data were downloaded and cleaned by removing ineligible and incomplete data, including “honesty” question failures. Data from the 66 trials with 12 target adverbs were extracted from the survey's dataset and, separately for each group (Australian bilinguals and monolinguals in Study 1; Australian and Russian bilinguals in Study 2), submitted to exploratory multidimensional scaling (MDS) using the smacofIndDiff function in the smacof package in R, version 2.1-0. (Groenen and van de Velden, 2016) set for ordinal data and the indscal constraint (Segalowitz et al., 2016). The MDS configuration of adverbs was then analyzed using hierarchical cluster analysis. The cluster-pattern approach was used to identify patterns within the parts of the whole configuration, and each cluster was ranked according to its estimated weight (Hout et al., 2013; Ding, 2018).

To assess intragroup consensus for each speaker group, following Segalowitz et al. (2016), we applied C-MDS which is a better fit for averaged data. For the evaluation of model fit, we used Kruskal's stress (group Stress-1) and median squared correlation coefficient (RSQ). Stress-1 (square root of 1-R2) is a standard MDS “lack of fit” measure for the group solution. Values of the Stress-1 higher than 0.1 may indicate a lack of fit. RSQ is an index of fit measure that shows how well the MDS solution works for the data.

### Ethical considerations

Ethics approval was obtained from the Human Research Ethics Committee at Queensland University of Technology (Ethics approval number 2516). Participation was voluntary, and participants could withdraw at any time. Anonymity, confidentiality, and protection of data were guaranteed.

## Study 1

Segalowitz et al. (2016) demonstrated the utility of the analytical approach in mapping semantic understanding and showed how Australian monolinguals represent epistemic adverbs. In this study, in a partial replication, we aim to confirm their results and, importantly, explore whether Russian-English bilinguals in Australia (Australian bilinguals) who speak Russian as their L1 and English as their L2 have the same understanding of the English epistemic adverbs as Australian monolinguals. To do so, we assessed intragroup consensus within the groups and determined the intersubjective normative cultural representation of the selected epistemic adverbs for each of our speaker groups. The recruitment procedures, materials, and methods were as described above.

## Results

The data for Study 1, after all exclusions, consisted of 83 valid questionnaires, 62 pertaining to Australian monolinguals and 21 pertaining to Australian bilinguals. Recall that among the exclusion criteria was a proficiency measure, where those who judged their proficiency in speaking and understanding English as less than 3 (on a 5-point scale, with 5 = *fluent ability*) were excluded from analysis, as well as those who indicated they spent less than 50% of their time in an English-speaking environment.

### Individual differences and intra-group consensus

Intersubjective norms, in contrast to statistical norms, are reflections of shared assumptions of members of a certain group about the values, beliefs, preferences, and behaviors of most members in the group or the culture of the group (Wan and Chiu, 2009). The research by Segalowitz et al. (2016) applied an aggregation method that took into account individual differences in knowledge of this consensus which was framed by cultural consensus theory (Romney et al., 1986). The cultural consensus approach supplements the classical MDS (C-MDS) to analyze data that aggregates the responses of participants to questions on epistemic adverbs based on their shared cultural knowledge.

Culture consists of shared cognitive representations of this structure, and the purpose of Cultural Consensus Theory (CCT) is to establish to which extent individuals in a group agree on these cognitive representations (Romney et al., 1996; Lacy et al., 2018). If no subgroups are identified, CCT models allow the assessment of each person's input or knowledge of the consensual representation. Measures of individual differences in this knowledge can then be used to more accurately characterize the group consensus with consideration of these individual differences when using C-MDS (Segalowitz et al., 2016).

Consensus analysis is similar to simple aggregation techniques, but it extends beyond them by providing a confidence level for each answer. Additionally, the informal cultural consensus model is also participant-oriented, meaning that it focuses on differences between participants, not variables (Weller, 2007; Baer et al., 2014). The CCT model first estimates individual competencies and then estimates the answers and the confidence in each answer. An individual is asked a series of questions requiring multiple-choice-type responses. The factor loadings are the estimated individual competence values, and the factor scores are the estimated answers: they constitute weighted, aggregated responses (Weller, 2007). First, these scores can be used to provide a metric to eliminate those participants whose answers differ significantly from the group consensus from the analysis. Second, they can be used as weights in the computation of the group aggregated data during C-MDS analysis, participants with higher factor loadings contribute to the group average more strongly than participants with lower loadings. As a result, C-MDS paired with cultural consensus analysis allows us to assess a cultural intersubjective norm while still considering individual variation in this consensus (Segalowitz et al., 2016). Importantly, the focus of the analyses is the adverbs themselves and how each adverb is understood (relative to each other adverb) by a speaker group.

Cultural competence estimates were provided by minimum residuals factor analysis (Weller, 2007; Segalowitz et al., 2016) using the fa function in the psych package in R (v. 1.2.5033). Research shows that replication is difficult when there are fewer than five or six salient variables (rows) per factor (column) (Gorsuch, 2015). The number of similarity judgments (rows) was 66, and we had 62 and 21 participants in the Australian monolingual and bilingual groups. We followed Segalowitz et al.'s (2016) approach to the calculation of the cultural competence estimates, according to which the factor analysis should include no more than 13 participants at a time. We resolved this by factor analyzing a randomly selected subset of 10 participants at a time (a ratio of approx. 7:1), repeating the procedure 1,000 times, and retaining median values from these 1,000 repeats. Participants' factor loadings were used as weights in computing a dissimilarity matrix with a group-level weighted average: participants with higher factor loadings contributed more than participants with lower loadings. This single matrix of aggregated data was then analyzed using the SmacofSym function of the smacof package in R, which performs a C-MDS analysis (see also Segalowitz et al. (2016)).

### Australian monolinguals

#### Consensus analysis

The existence of a group consensus among Australian monolinguals was supported by a ratio of first-to-second factor eigenvalues >3.0. We obtained a ratio of 9.167 (the ratio of first:second eigenvalues = 3.93:0.429). The median competence score was 0.650 (median absolute deviation (MAD) = 0.143), above the recommended 0.50 average (Weller, 2007), confirming that there was consensus in the representation of the adverbs of certainty and uncertainty. Competence scores below 0.30 were considered a significant departure from consensus (Weller, 2007). In our sample, five participants had competence scores under 0.30 and were eliminated, resulting in 57 participants for analysis. Competence scores were then used as weights in a group-level weighted average dissimilarity matrix.

#### Statistical acceptability of the weighted-data C-MDS results

Table 2 reports model fit values for both 2D and 3D solutions. Following Segalowitz et al. (2016), we reported fit values for both weighted and unweighted analyses (i.e., with no adjustment by factor loadings, using simple mean aggregation). The use of weighted data improved the model fit over unweighted data, with the weighted data yielding higher RSQ and lower Stress-1 values for the 3D solution which supports the use of consensus analysis. Kruskal and Wish (1978) suggested using Stress-1, RSQ values, and an interpretation of configuration for the selection of appropriate dimensionality. Stress-1 values below 0.05 were considered excellent, between 0.05 and 0.10 were good, between 0.10 and 0.20 were fair, and above 0.20 were poor (Kruskal and Wish, 1978).

**Table 2:** Model fit results for Australian monolinguals using MDS with and without weighted data derived from cultural consensus analysis.

**Model**	**Stress-1**	**RSQ**
**3-dimensional solution**		
Weighted data	0.004	0.779
Unweighted data	0.003	0.664
**2-dimensional solution**		
Weighted data	0.041	0.810
Unweighted data	0.016	0.811

For the 2D solution, unweighted data yielded a slightly higher RSQ, however, the difference in RSQ between 3D and 2D was insignificant (difference = 0.0009). All further results referred to the weighted data analyses. Although fit indices for the weighted analysis for both the 3D solution (Stress-1 = 0.004, RSQ = 0.779) and 2D solution (Stress-1 = 0.041, RSQ = 0.810) were robust, for our Australian monolinguals, we chose a 3D solution in line with Segalowitz et al. (2016). Accordingly, the semantic analyses that follow were based on the 3D solution.

#### Semantic analysis

Interpretation of the output of cluster analysis with MDS can be subjective (Guest, 2013). To aid interpretation, the coordinates for each adverb, taken from the group solution in the weighted-data C-MDS analysis, were submitted to hierarchical cluster analysis, using the R package “fpc” with cluster method = hclustCBI, method = ward.D2, k = 4, and 100 bootstrap replications (Segalowitz et al., 2016). The bootstrap is used to get an idea of bias and variation caused by the chosen statistical method (Hennig, 2007). For the bootstrap scheme, a valid, stable cluster should yield a Jaccard similarity value of 0.75 or more. Between 0.6 and 0.75, clusters may be considered as indicating patterns in the data. “Highly stable” clusters should yield Jaccard similarity values of 0.85 and above (Henning, 2020).

**Figure 1:**
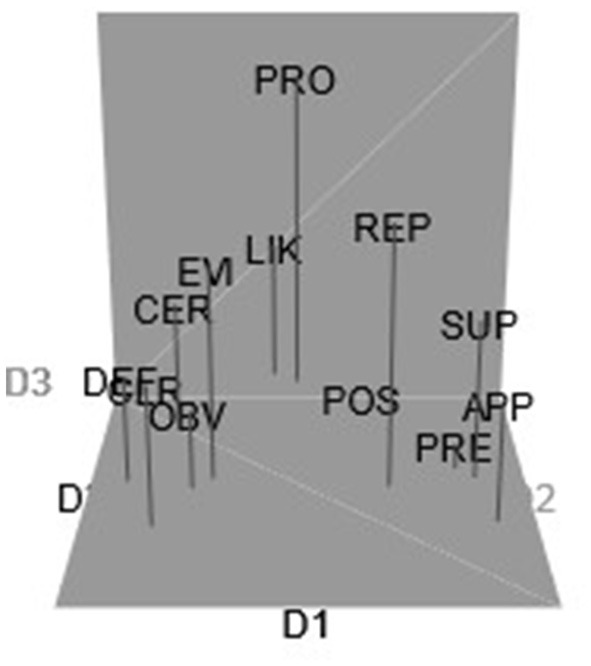
Configuration of target epistemic adverbs in the Australian monolingual sample (3D). APP, apparently; CER, certainly; CLR, clearly; DEF, definitely; EVI, evidently; LIK, likely; OBV, obviously; POS, possibly; PRE, presumably; PRO, probably; REP, reportedly; SUP, supposedly.

Figure 1 shows the clustering patterns that appeared from the semantic analysis. The analysis revealed the following clusters for the Australian monolinguals (including their respective Jaccard similarity values):

CLUSTER i: *Apparently, Presumably, Reportedly, Supposedly* (Jaccard index = 0.816)
CLUSTER ii: *Certainly, Clearly, Definitely, Evidently, Obviously* (Jaccard index = 0.876)
CLUSTER iii: *Likely, Possibly* (Jaccard index = 0.682)
CLUSTER iv: *Probably* (Jaccard similarity index = 0.585)

Figure 1 shows the contrast between adverbs expressing higher levels of confidence (*certainly, clearly, definitely, obviously*, and *evidently* on the left side of the plot along D1; Cluster ii) vs. adverbs with lower levels of confidence (Cluster i: *apparently, presumably, reportedly, supposedly)*, on the right side of the plot along D2. The adverbs on the right side refer to the speaker's information source and convey information at some “distance” from the speaker. Cluster iii contains *likely* and *possibly*. Adverbs in Clusters iii and iv (*likely, possibly*, and *probably*) are likelihood adverbs, and it is interesting to see that participants clustered *likely* and *possibly* together, while *probably* constituted a separate cluster (Cluster iv). A higher level of objectivity associated with *probably* could be a plausible explanation for a separate cluster for this adverb. We should also note that clusters with Jaccard values between 0.6 and 0.75 are only indications of patterns, while clusters with low Jaccard values (below 0.6) are considered unstable. Cluster iv (Jaccard value = 0.585) is unstable, so the results are only indicative.

### Russian-English bilinguals in Australia

#### Consensus analysis

The existence of a group consensus among Australian bilinguals was supported by a ratio of first-to-second factor eigenvalues >3.0. If the ratio is 3 to 1 or larger, then the consensus model may be used to represent the group's responses with a single set of answers (Weller, 2007). We obtained a ratio of 11.346 (the ratio of first:second eigenvalues = 4.519:0.398). The factor loadings on the 1-factor solution provided individual cultural competence scores indexing the degree to which each participant's data correlated with the factor (Weller, 2007). The median competence score was 0.724 (MAD = 0.093), above the recommended 0.50 average (Weller, 2007), confirming that there was a consensus in the representation of the adverbs of certainty and uncertainty. One participant had a competence score below 0.30 and was excluded from further analysis, resulting in 20 participants whose ratings were retained for analysis. These results indicate a consensus among Australian Russian-English bilinguals in their response to the epistemic adverbs. Competence scores were then used as weights in a group-level weighted average dissimilarity matrix.

#### Statistical acceptability of the C-MDS results

Table 3 shows the model's fit values for both 2D and 3D solutions obtained with the C-MDS analysis using unweighted data and weighted data based on the factor loadings from the consensus analysis. As can be seen in Table 3, both weighted and unweighted data yielded excellent Stress-1 values (<0.05). For the 3D solution, weighted data yielded a higher RSQ (0.549) than for the 2D solution (0.493). All further results refer to the weighted data analyses. Although the minimum recommended value for RSQ is 0.60, research on multiple regression in L2 studies shows that the median R2 value for such studies is 0.32, thus it explains approximately one-third of the variance. Thereafter, we might consider it as fairly robust model that explains roughly 50% or more of the variance relative to the studies in L2 (Plonsky and Ghanbar, 2018). The semantic analyses below are based on the 3D solution.

**Table 3:** Model fit results for the Australian bilinguals using MDS with and without weighted data derived from cultural consensus analysis.

**Model**	**Stress-1**	**RSQ**
**3-dimensional solution**		
Weighted data	0.010	0.549
Unweighted data	0.005	0.489
**2-dimensional solution**		
Weighted data	0.047	0.493
Unweighted data	0.038	0.491

#### Semantic analysis

Our analysis revealed the following four clusters (including their respective Jaccard similarity values):

CLUSTER 1: *Apparently, Presumably, Supposedly* (Jaccard index = 0.794)
CLUSTER 2: *Certainly, Clearly, Definitely, Obviously* (Jaccard index = 0.903)
CLUSTER 3: *Evidently, Probably, Reportedly* (Jaccard index = 0.871)
CLUSTER 4: *Likely, Possibly* (Jaccard index = 0.644)

**Figure 2:**
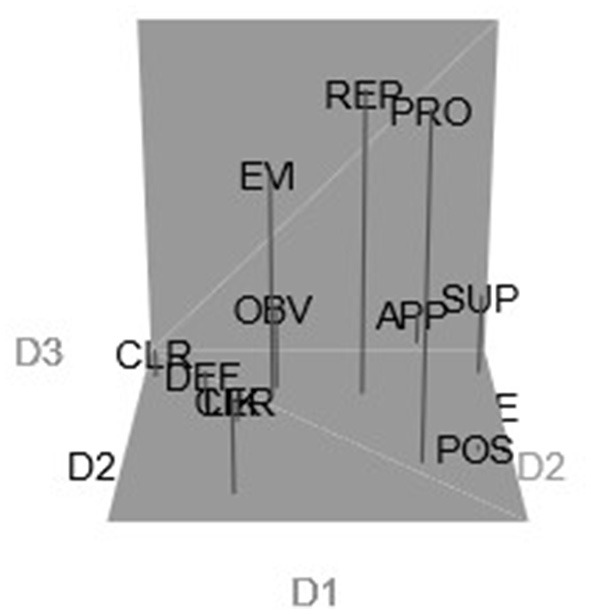
Configuration of target epistemic adverbs in the Australian bilingual sample (3D). APP, apparently; CER, certainly; CLR, clearly; DEF, definitely; EVI, evidently; LIK, likely; OBV, obviously; POS, possibly; PRE, presumably; PRO, probably; REP, reportedly; SUP, supposedly.

Cluster 2 consists of “confident” adverbs (*certainly, clearly, definitely, obviously)*. This is the tightest cluster, with a Jaccard similarity value of 0.903. Figure 2 shows the contrast between adverbs with a high level of confidence on the left side of the plot, along D1 (Cluster 2), and the adverbs with a meaning which is distant from the speaker (*apparently, presumably, supposedly*) on the right side of the plot, along D2 (Cluster 1, Jaccard similarity value = 0.794). Interestingly, Cluster 3 includes *evidently, probably*, and *reportedly* (Jaccard similarity value = 0.871). While adverbs *evidently* and *reportedly* refer to the logical evaluation, *probably* is based on the subjective attitude of the speaker. This could be explained by the influence of the participants' L1. Finally, Cluster 4 has two likelihood adverbs, *likely* and *possibly* (Jaccard similarity value = 0.644) with a higher level of uncertainty. Overall, the results showed a strong community consensus, low Stress-1, medium RSQ, and generally stable and interpretable semantic outcomes.

## Preliminary discussion

For the Australian monolinguals, C-MDS analysis returned statistically acceptable results with a high RSQ value for the 3D model with weighted means (RSQ = 0.779) and a low level of stress (Stress-1 = 0.004). There was evidence for consensus between participants supported by a ratio of first-to-second factor eigenvalues >3.0 (9.167). Of particular interest is whether our monolingual speaker group understood the same epistemic adverbs, used in the identical health context (paired medical opinions), in the same way as the speaker group studied by Segalowitz et al. (2016).

### Comparing Australian monolinguals

Our findings are consistent with those reported by Segalowitz et al. (2016). Specifically, Cluster i in our data (*apparently, presumably, reportedly, supposedly*) is identical to the Cluster 1 they reported for their Australian monolinguals. This cluster conveys a personal stance about a source of knowledge that is distant from a speaker. Based on the results from both studies, it appears that native, monolingual speakers of Australian English clearly distinguish adverbs which explicitly indicate indirect knowledge.

However, we did observe some differences related to “confident”/“certain” adverbs and adverbs with lower confidence which convey information at a certain distance from a speaker (Cluster ii vs. Cluster i). For our monolinguals, *evidently* was included in Cluster ii, with the other high confidence adverbs (c*ertainly, clearly, definitely, obviously*). In a similar group of speakers, Segalowitz et al. (2016) found that *evidently* was clustered with *likely* and *obviously*. In our data, participants considered both *evidently* and *obviously* as “confident” adverbs. The slight variation in clustering patterns between the two speaker groups suggests nuanced differences in how “confidence” adverbs are understood. Cinque (1999) and Simon-Vanderbergen and Aijmer (2007) consider *evidently* as an evidential adverb rather than an epistemic one. As the name suggests, *evidently* has a semantic component of evidentiality. At the same time, it also conveys a certain level of conviction. Similarly, *obviously* conveys both certainty and confidence. Biber (2006) included *obviously* into the group of “certain” adverbs, along with *certainly* and *definitely*. These nuances in the meanings of *evidently* and *obviously* could explain their inclusion into the “certainty” cluster for our Australian monolinguals.

Although the Jaccard similarity indices for the remaining Clusters iii and iv are not high, they are still indicative. In our data, *probably* was clustered separately (Cluster iv) from *likel*y and *possibly* (Cluster iii). Segalowitz et al. (2016), for a comparable speaker group, reported that *possibly* and *probably* were clustered together, while *likely* was clustered with *evidently* and *obviously*. Although none of these three adverbs (*likely, possibly*, and *probably*) convey any information about the source, they do convey a different level of uncertainty, with *probably* having a higher level of certainty than *possibly* and *likely*. There have even been attempts to assign numeric values to these adverbs when used in health communication, such as 75% for *probably*, 50% for *possible*, and 25% for *less likely* (Panicek and Hricak, 2016); however, there is no unanimous agreement among researchers on the specific numeric values for epistemic adverbs.

Despite some variations, native speakers of Australian English clearly distinguish between two groups of epistemic adverbs: adverbs of confidence/certainty and adverbs with a distant source of information. The differences between our monolinguals and those studied by Segalowitz et al. (2016) are interesting and may reflect the following. First, language is anything but static, and aspects of language use and the meaning assigned to words constantly change. Analysis of vernacular speech data collected in the UK and Canada over the past 30 years showed changes in the use of such adverbs as *clearly, evidently, obviously*, and *of course*. For example, in both Canada and the UK, people born in 1990-2001 use *obviously* significantly more frequently than people born in 1931-1960 (Tagliamonte and Smith, 2021), suggesting differences between speaker groups that may be reflective of their experiences and how those are coded linguistically. Segalowitz et al.'s (2016) study was conducted in 2014, while data for our study were collected in 2020, amid the COVID pandemic. Our monolingual speakers of Australian English may use and understand epistemic adverbs somewhat differently from a comparable group 6 years earlier, perhaps because of how risk and uncertainty were discussed at that time. Second, there are different forces that drive language changes, including group identity. Trask (2009) noted that just like a group can be identified by its members' clothes or hairstyles, it can also be identified by their speech. The group identity of our monolingual participants was different in terms of their level of education, which may have impacted the meaning nuances attributed to epistemic adverbs. While participants in Segalowitz et al.'s (2016) study all were first-year Psychology students, a third of our monolingual speaker group included participants with undergraduate (n = 17) and postgraduate degrees (n = 2). We should also note that neither our Study 1 nor Segalowitz et al. (2016) Study 1 included older Australians. Out of interest, we ran our C-MDS that included our older monolinguals (n = 5), and the outcome differed. (A follow-up study is underway in which we explore how age may affect one's semantic coding of epistemic adverbs).

### The L1 effect: comparing Australian bilinguals and monolinguals

For the Australian Russian-English bilinguals, the C-MDS analysis returned statistically acceptable results with a moderate level of variance accounted for by the RSQ value for the 3D model with weighted means (RSQ = 0.549) and the low level of stress (Stress-1 = 0.01). There was evidence for consensus between participants supported by a ratio of first-to-second factor eigenvalues >3.0 (11.34). Of interest is whether the Australian bilinguals understood the epistemic adverbs similarly to the monolinguals. Both groups resided in Australia, and the bilinguals professed a high level of proficiency in, and use of, English.

**Figure 3:**
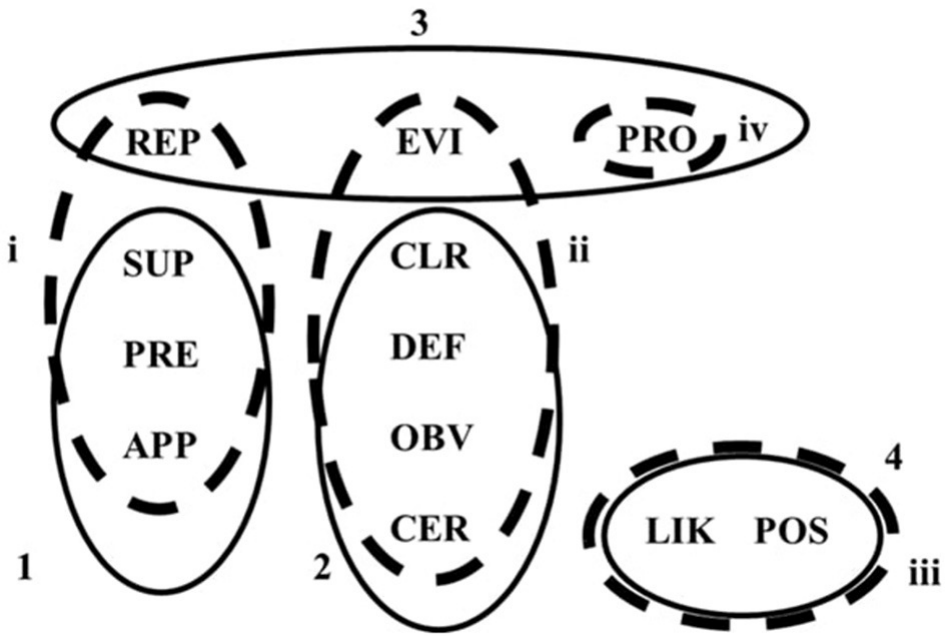
Semantic understanding of epistemic adverbs by Australian bilinguals and Australian monolinguals. Australian bilinguals (solid line); Cluster 1 (Jaccard value = 0.794); Cluster 2, Jaccard value = 0.903; Cluster 3, Jaccard value = 0.871; Cluster 4, Jaccard value = 0.644. Australian monolinguals (dashed line); Cluster i, Jaccard value = 0.816; Cluster ii, Jaccard value = 0.876; Cluster iii, Jaccard value = 0.682; Cluster iv, Jaccard value = 0.585. APP, apparently; CER, certainly; CLR, clearly; DEF, definitely; EVI, evidently; LIK, likely; OBV, obviously; POS, possibly; PRE, presumably; PRO, probably; REP, reportedly; SUP, supposedly.

As can be seen in Figure 3, there were both similarities and differences in the comprehension of adverbs of certainty and doubt by the Australian bilinguals and monolinguals. The observed similarities in the clustering of epistemic adverbs are likely to have emerged because of the dominant and shared, language environment (Australian English) and the high level of exposure to (and use of) English. For example, the two speaker groups distinguished confidence adverbs from other adverbs, clustering *certainly, clearly, definitely*, and *obviously* together (see the overlap between Cluster 2 and Cluster ii; Figure 3). They only differed in how *evidently* was understood. While the monolinguals included *evidently* with the certainty adverbs, the bilinguals clustered it with *probably* and *reportedly* (Cluster 3). This slight deviation in how the Australian bilinguals understood *evidently* (within the context of medical opinions) may be explained by the fact that this adverb does not have a direct equivalent in Russian. Instead, its partial equivalents refer to the logical evaluation of the validity of the utterance rather than a speaker's subjective attitude.

The Australian bilinguals and monolinguals were also largely in agreement regarding their understanding of hearsay adverbs, particularly *apparently, presumably*, and *supposedly* (see the overlap between Cluster 1 and Cluster i; Figure 3). Interestingly, for Australian monolinguals, the hearsay cluster (Cluster i) also included *reportedly*. *Apparently, presumably, reportedly*, and *supposedly* have the following semantic component: something a person heard of or read about (Wierzbicka, 2006), and this appears to be the basis of the monolinguals' understanding of these adverbs. In contrast, the Australian bilinguals clustered *reportedly* with *probably* and *evidently* (Cluster 3; see Figure 3), a very stable cluster (Jaccard index = 0.871). The overlap in the understanding of *reportedly, probably*, and *evidently* by Australian bilinguals may be explained by the fact that *reportedly* has been added to the class of epistemic adverbs relatively recently and is associated with the increasing role of media in contemporary society (Wierzbicka, 2006). It is conceivable that our Russian-English speakers in Australia have had comparatively less exposure to the Australian media because they may consume media in Russian. We note that, nationally, news in Russian, Russian radio, and social media groups operate daily.

Both groups shared the same understanding of the likelihood adverbs, *likely* and *possibly* (see the complete overlap between Cluster 4 and Cluster iii; Figure 3). It is worth noting that for the monolinguals, *probably* formed a unique cluster. However, it was also the weakest one and the least reliable (Jaccard value = 0.585). Results of a vignette-based study on the interpretation of uncertainty expressions conducted in the US showed that young patients (adolescents) and HPs understood several epistemic expressions, including the epistemic adverb *probably*, differently from each other (Cohn et al., 1995). Importantly, in their study, the difference in understanding was based not only on the participants' role, patient or HP, but also on age (which was confounded with the participants' role). This suggests not only that the understanding of *probably* may vary depending on the speaker group, but also depending on the age of the speakers.

## Study 2

One possibility raised by the results of Study 1, when compared with Segalowitz et al.'s (2016) Study 1, is that language context (including the degree of exposure to and use of another language) may impact one's understanding of words, such as epistemic adverbs. The aim of Study 2, therefore, was to determine whether differences in exposure to and use of L2, English, impact the comprehension of English epistemic adverbs. We explored this question by comparing the dissimilarity ratings of the Russian-English bilinguals based in Australia (Australian bilinguals) with those provided by Russian-English bilinguals based in Russia (Russian bilinguals). Materials and procedures were followed as described in Study 1.

### Russian-English bilinguals in Russia

#### Consensus analysis

The existence of a group consensus among Russian-English bilinguals in Russia was supported by a ratio of first-to-second factor eigenvalues >3.0. We obtained a ratio of 7.955 (the ratio of first:second eigenvalues = 4.275:0.537) and strong cultural competence scores, with a median competence score of 0.69 (MAD = 0.139, above the recommended 0.50 average (Weller, 2007)). One participant had a competence score below 0.30 and was excluded, resulting in 16 participants for further analysis. These results indicate a consensus among Russian bilinguals in their response to the epistemic adverbs.

#### Statistical acceptability of the C-MDS results

Table 4 reports model fit values for both 2D and 3D solutions obtained with the C-MDS analysis using unweighted data and weighted data based on the factor loadings from the consensus analysis for the Russian bilinguals. As shown in Table 4, both weighted and unweighted data yielded excellent Stress-1 values (<0.05). For the 2D solution, weighted data yielded a higher RSQ (RSQ = 0.52) than for the 3D solution (RSQ = 0.44). We chose the 2D solution, because of the better fit results for the weighted analysis for the 2D solution (Stress-1 = 0.03 and RSQ = 0.52) compared to the 3D solution (Stress-1 = 0.005, RSQ = 0.44). Like the Australian bilinguals, the Russian bilinguals responded to the survey in their L2 (English), the difference being that their reported use of L2 was much lower (median daily use of English = 30%), reflecting the fact that they lived and worked in Russia. The semantic analysis below is based on the 2D solution.

**Table 4:** Model fit results for Russian bilinguals using MDS with and without weighted data derived from cultural consensus analysis.

**Model**	**Stress 1**	**RSQ**
**3-dimensional solution**		
Weighted data	0.005	0.440
Unweighted data	0.005	0.506
**2-dimensional solution**		
Weighted data	0.028	0.523
Unweighted data	0.041	0.343

#### Semantic analysis

**Figure 4:**
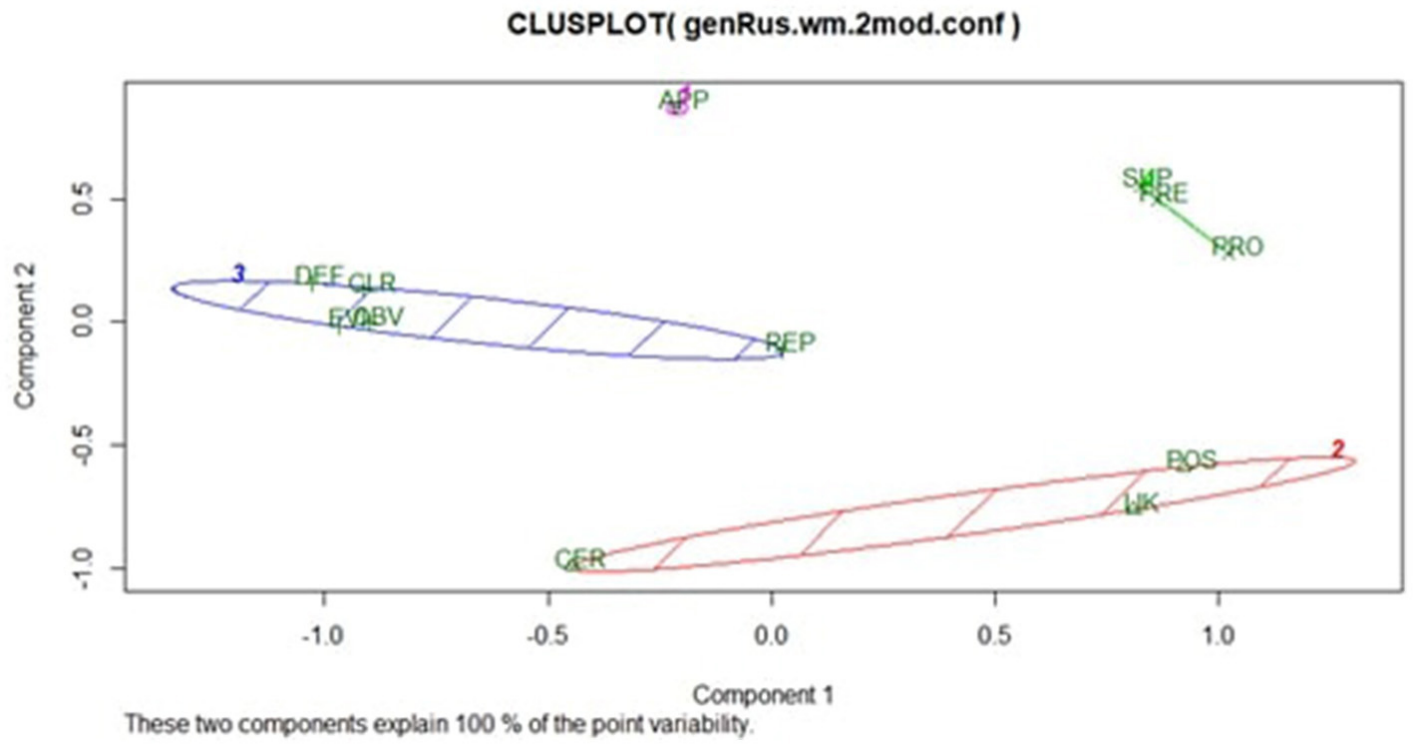
Configuration of target epistemic adverbs in the Russian bilingual sample (2D). APP, apparently; CER, certainly; CLR, clearly; DEF, definitely; EVI, evidently; LIK, likely; OBV, obviously; POS, possibly; PRE, presumably; PRO, probably; REP, reportedly; SUP, supposedly.

Figure 4 shows the 2D configuration yielded by the weighted C-MDS analysis and reports the results of hierarchical cluster analysis using the fpc package. The analysis revealed the following clusters:

CLUSTER i: *Apparently* (Jaccard index = 0.533)
CLUSTER ii: *Certainly, Likely, Possibl*y (Jaccard index = 0.764)
CLUSTER iii: *Clearly, Definitely, Evidently, Obviously, Reportedly* (Jaccard index = 0.869)
CLUSTER iv: *Presumably, Probably, Supposedly* (Jaccard index = 0.859)

Cluster iii, which includes the “confident” adverbs *clearly, definitely, evidently, obviously*, and *reportedly*, is the tightest cluster. *Presumably, probably*, and *supposedly* formed another tight cluster (Cluster iv). Neither *presumably* nor *supposedly* have direct equivalents in Russian; their translations depend on the context. In English, there are also low frequency adverbs (Wierzbicka, 2006), which may explain their clustering with *probably*, the most common adverb out of these three. Interestingly, the adverb *apparently* formed a separate cluster (Cluster i). However, it is also the weakest cluster, suggesting that the understanding of *apparently* by Russian bilinguals is only indicative.

Finally, Cluster ii includes *certainly, likely*, and *possibly*. While the Russian equivalents of *certainly*, the non-epistemic adverbs непременно (*nyepryemyenno*) and обязательно (*obyazatelno*), both have strong semantic components of certainty with no likelihood meaning, it is conceivable that the Russian bilinguals' interpretation of *certainly* was based mostly, if not entirely, on their formal knowledge of L2, English. Given that these Russian-based participants were predominantly university educated (see Table 1), their experience with English is likely to have been mostly through reading. In English, *certainly*, despite having a strong lexical component of “certainty,” is classified as an epistemic adverb, meaning that a speaker does not have full knowledge or is unsure about having the knowledge (Wierzbicka, 2006). The lexical component reflecting incomplete knowledge, which *certainly* has in common with the likelihood adverbs *likely* and *possibly*, may explain why Russian bilinguals understood these three adverbs similarly.

## Preliminary discussion

For the Russian bilinguals (Russian-English speakers in Russia), MDS analysis returned statistically acceptable results with a moderate level of variance accounted for by the RSQ value for the 2D model with weighted means (RSQ = 0.523) and the low level of stress (Stress-1 = 0.028). There was evidence for consensus between participants, supported by a ratio of first-to-second factor eigenvalues >3.0 (7.955).

The meaning structure revealed by MDS analysis was interpretable. Differences in fit results, namely the 2D solution for the Russian bilinguals and the 3D solution for the Australian bilinguals, may be explained by the varied level of exposure to English between the groups. While the Australian bilinguals use English daily (i.e., at least 50% of the time) and in different contexts, because the Russian bilinguals live and work in Russia, they do not have the same level of exposure (i.e., median daily use of English = 30%). The fact that the 2D solution shows a better fit for Russian bilinguals may reflect their less nuanced understanding of the meanings of epistemic adverbs in English.

**Figure 5:**
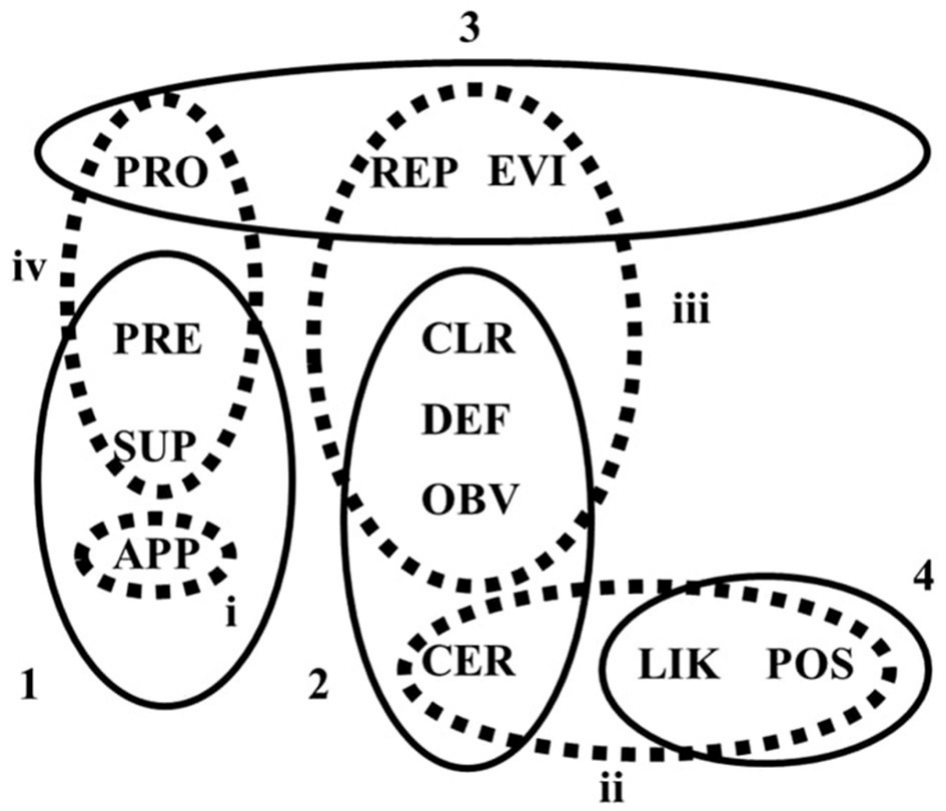
Semantic understanding of target epistemic adverbs by Russian-English bilinguals depending on their context (Australia vs. Russia). Australian bilinguals (solid line); Cluster 1 (Jaccard value = 0.794); Cluster 2, Jaccard value = 0.903; Cluster 3, Jaccard value = 0.871; and Cluster 4, Jaccard value = 0.644. Russian bilinguals (dashed line); Cluster i (Jaccard value = 0.533); Cluster ii, Jaccard value = 0.764; Cluster iii, Jaccard value = 0.869, and Cluster iv, Jaccard value = 0.859. APP, apparently; CER, certainly; CLR, clearly; DEF, definitely; EVI, evidently; LIK, likely; OBV, obviously; POS, possibly; PRE, presumably; PRO, probably; REP, reportedly; SUP, supposedly.

The semantic analysis revealed some interesting similarities and differences in the understanding of epistemic adverbs between Australian and Russian bilinguals (see Figure 5). Although we cannot directly compare the results of the two bilingual speaker groups because of the differences in the nuanced understanding, as reflected in the 3D vs. 2D solutions, we can make some observations. For example, with respect to the “confident” adverbs, the Russian bilinguals clustered *evidently, clearly, definitely, obviously*, and *reportedly* in a broad grouping (Cluster iii, Jaccard value = 0.869; see Figure 5), while Australian bilinguals showed a tighter cluster that also included *certainly* but excluded *evidently* and *reportedly (certainly, clearly, definitely*, and *obviously)* (Cluster 2, Jaccard value = 0.903). Our two bilingual speaker groups also placed *probably* in different clusters. The Australian bilinguals clustered it together with *evidently* and *reportedly* (Cluster 3), while the Russian bilinguals understood its meaning to resemble that of *presumably* and *supposedly* (Cluster iv). For the Russian bilinguals, the semantic component of uncertainty determined how *probably* was represented semantically.

The above observations suggest that the differences seen between the two bilingual groups reflect the differences in their exposure to and use of English. Specifically, our Australian bilingual speakers, who reside in a country that is monolingual English-speaking, showed a greater nuance of meaning, as demonstrated by the 3D solution and more reliable clusters. Our findings are consistent with Isurin's (2007) observations of the impact of the dominant L2 of the country of residence on one's language use. Analysis of speech samples in L1, Russian, elicited from Russian-English bilinguals residing in the USA, revealed a much greater number of lexical borrowings and syntax deviations compared to speech samples taken from Russian monolinguals in Russia. These differences were found even though the Russian-English bilinguals in Isurin's (2007) study were Russian language instructors working at US Universities who used their L1 (Russian) also professionally. Australian bilinguals who do not use Russian professionally are likely to experience an even greater influence of L2 (English). Their immersion in English and its use in daily life is what has contextualized and determined their understanding of English (L2) words, in this case, epistemic adverbs. As seen in Study 1, Russian bilinguals clustered certainty adverbs differently (*clearly, definitely, evidently, obviously*, and *reportedly*) which may be explained by their immersion in the L1 (Russian) environment and their lower exposure to English (L2).

## General discussion

We examined how Russian-English bilingual speakers comprehend English adverbs of certainty and doubt when used in health communication. Specifically, in Study 1, we explored whether, and if so how, the understanding of these epistemic adverbs by Russian-English bilinguals resident in Australia (Australian bilinguals) differed from that of Australian monolingual English speakers. In Study 2, we explored to what extent the understanding revealed for Australian bilinguals (which was subtly different from their monolingual peers) resembled that of a Russian-English bilingual speaker group in Russia. If their understanding was impacted solely by the knowledge of Russian as their L1, the bilinguals would be expected to share common understandings. Importantly, we constrained the context within which the English epistemic adverbs were presented by embedding them in pairs of doctor's opinions that differed only in the epistemic adverb used. Participants were asked simply to rate how dissimilar in meaning the pairs of sentences were. The resulting dissimilarity ratings were analyzed with the help of MDS scaling, cultural consensus, and cluster analyses. Goodness-of-fit values, semantic space solutions, stress decomposition analysis,^3^ and stability of clusters in all sets of data indicated both similarities and differences between the Australia-based bilinguals and the monolingual speakers of Australian English (Study 1), as well as between the bilingual speaker groups in Australia and Russia (Study 2).

The use of C-MDS, supplemented by cultural consensus and cluster analyses, helped to reveal whether bilingual Russian-English speakers share the same understanding of English epistemic adverbs as monolingual speakers of Australian English. It also helped to identify similarities and differences in the comprehension of English epistemic adverbs used in health communication between groups of bilingual Russian-English speakers based on their language context and exposure to L2 (English).

Several findings are particularly important for understanding similarities and differences in lexical comprehension between bilingual Russian-English speakers and monolingual speakers of Australian English. First, both bilingual groups and our monolingual group clustered adverbs of “confidence”/“certainty” into a separate cluster (Cluster iii for Australian monolingual speakers and Clusters 2/ii for both bilingual groups), contrasting them with adverbs of doubt (refer to Figures 3 and 5). This observation is reassuring, especially within a health context, because it demonstrates that there is no language-dependent ambiguity for certainty adverbs in English (at least not for L1 speakers of Russian).

Second, our findings show that the level of exposure to L2 can influence the interpretation of English epistemic adverbs by Russian-English bilinguals. For example, Australian bilinguals clustered *apparently, presumably*, and *supposedly* together, just like our monolinguals (who included *reportedly* in this cluster as well). Australian monolinguals and bilinguals also shared a similar understanding of high confidence adverbs (*certainly, clearly, definitely*, and *obviously*); however, the Australian monolinguals understood *evidently* similarly, and it featured as an additional high confidence adverb. *Evidently* was also interpreted differently by Australian and Canadian monolinguals in Segalowitz et al.'s (2016) Study 1. This difference in understanding may reflect different interpretations of the nature of *evidently*, while Wierzbicka (2006) placed *evidently* into the group of epistemic adverbs, Cinque (1999) called it evidential (along with *allegedly, reportedly, apparently, obviously*, and *clearly*) as opposed to speaker-oriented (*probably, likely, presumably*, and *supposedly*). This disparity in linguistic analyses suggests that speakers in different communities and age groups may consider *evidently* as either evidential or epistemic, and any such difference in their understanding should emerge from a cluster analysis using the type of paradigm and analytical approach applied herein.

Third, the Australian bilinguals resembled the Australian monolinguals more closely in their understanding of epistemic adverbs than the Russian bilinguals. For example, like the monolinguals, they understood *apparently* to be close in meaning to the other hearsay adverbs, whereas the Russian bilinguals appeared uncertain, as revealed by the singular, unstable cluster (low Jaccard similarity value = 0.533). Similarly, while Australian bilinguals and monolinguals clustered adverbs of likelihood *likely* and *possibly* together, Russian bilinguals also included *certainly*, reflecting a slightly different interpretation of these adverbs and one that may be driven by the use of English that is predominantly receptive (i.e., reading).

## Conclusion

Our results revealed similarities and differences in the comprehension of the target epistemic adverbs both between the bilingual and monolingual speaker groups, because of bilinguality, and between the two bilingual speaker groups, depending on their language context. Overall, Australian bilinguals showed more similarities in comprehension of the said adverbs to Australian monolinguals than Russian bilinguals. This suggests that a degree of exposure to and use of L2 plays an important role in the comprehension of English epistemic adverbs by bilingual speakers. However, the observed similarities are mostly related to the high confidence adverbs and the hearsay adverbs. Adverbs of doubt were interpreted somewhat differently, suggesting that this could be the case across other speech communities. Australian HPs need to be aware of these potential differences in the understanding of epistemic adverbs that reflect degrees of doubt and, when they use these themselves, ensure to check for understanding and provide additional or alternative explanations as appropriate.

## Practical implications

Our study has several practical implications. First, in health interactions, HPs should be aware of potential differences in understanding epistemic adverbs and reflect on their use. When HPs are bilingual themselves and, as our results suggest, especially when their L1 is Russian, they should double check the understanding of uncertainty by their English-speaking patients to ensure that this matches their own. This is particularly important when epistemic adverbs are used to communicate the uncertainty of diagnosis, prognosis, and/or treatment. Second, bilingual Russian-English patients also should be made aware and be mindful of differences in the comprehension of epistemic adverbs that may exist between them and their HP. They should be encouraged to seek clarification from their HP when in doubt or, particularly when their difficulties in understanding are a source of concern, request the assistance of an interpreter. We specifically selected our bilinguals to be more than moderately proficient in their L2 (English). It is therefore unknown how one's understanding may be impacted by a poorer command of the L2. Therefore, a final suggestion is that future research should explore the understanding of epistemic adverbs, and perhaps other epistemic expressions in the health context, as related specifically to differences in L2 usage and proficiency if L2 is the language used to discuss health concerns.

## Data availability statement

The raw data supporting this article cannot be made available because the ethics approval does not permit it. For any further enquiries, please contact the corresponding author.

## Ethics statement

The studies were reviewed and approved by the University Human Research Ethics Committee, Queensland University of Technology, Brisbane, Australia. The participants provided written consent.

## Author contributions

VN was responsible for the data collection and original analyses and led the development of the manuscript. RM provided research supervision. Both authors contributed to the conceptualization of the project and its methodology.

## Figures and Tables

**Figure A1:**
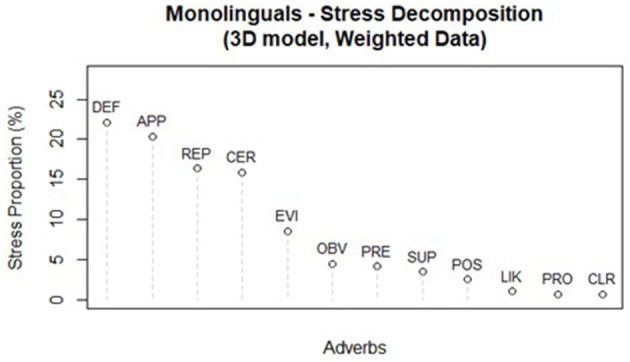
Stress decomposition per adverb (percent contribution to overall stress by each adverb) in the Australian monolinguals. APP, apparently; CER, certainly; CLR, clearly; DEF, definitely; EVI, evidently; LIK, likely; OBV, obviously; POS, possibly; PRE, presumably; PRO, probably; REP, reportedly; SUP, supposedly.

**Figure A2:**
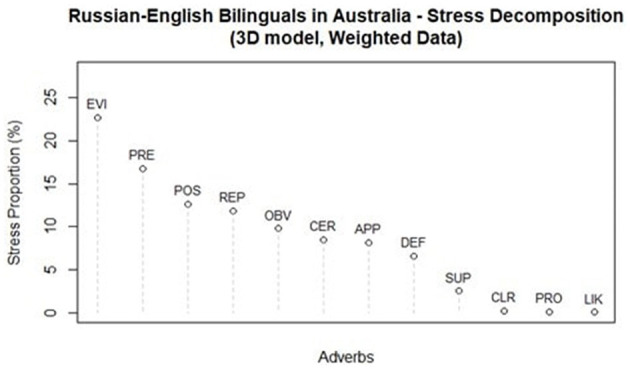
Stress decomposition per adverb (percent contribution to overall stress by each adverb) in the Russian bilinguals in Australia. APP, apparently; CER, certainly; CLR, clearly; DEF, definitely; EVI, evidently; LIK, likely; OBV, obviously; POS, possibly; PRE, presumably; PRO, probably; REP, reportedly; SUP, supposedly.

**Figure A3:**
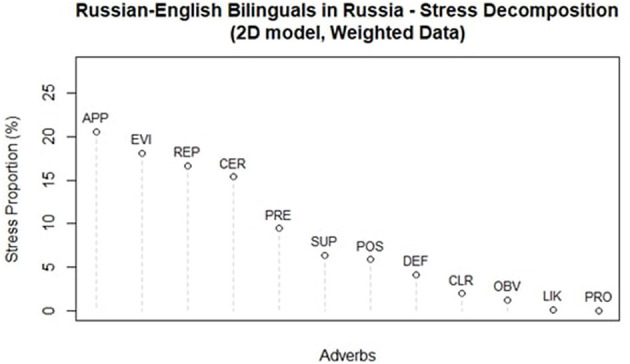
Stress decomposition per adverb (percent contribution to overall stress by each adverb) in the Russian bilinguals in Russia. APP, apparently; CER, certainly; CLR, clearly; DEF, definitely; EVI, evidently; LIK, likely; OBV, obviously; POS, possibly; PRE, presumably; PRO, probably; REP, reportedly; SUP, supposedly.

## Footnotes

^1^In labelling the bilinguals as Russian-English bilinguals, we follow the convention of naming L1 first (i.e., Russian) and then the second language (L2; English).

^2^The honesty question picked up if respondents were answering without reflection. It read: “If you answered the honesty questions in the survey carefully and honestly, please check the option “Other” and then type “Ok” into the box.”

^3^For results of stress decomposition analysis, please refer to Appendix 1.

## Appendix 1

### Stress decomposition analysis

Following Segalowitz et al. (2016), we also compared the data sets by decomposing Stress-1 values, looking at each adverb's stress-per-point (SPP) on a percentage scale. It gives us an idea of how much each adverb contributes to the fit (see Figures A1–A3). In both bilingual data sets, there were only two outliers in each set, with 10 of the 12 adverbs accounting for about or less than 15% of the stress and with many adverbs accounting for 5% or less.

For the Australian bilingual data set, the outliers were *evidently* = 24% and *presumably* = 17%. In the sample with Russian bilinguals, there were two adverbs with high SPP values: *apparently* (SPP = 21%), *evidently* (SPP = 18%), followed by *reportedly* (SPP = 17%), *certainly* (SPP = 16%). The similarity of the remaining values indicates that remaining adverbs equally contributed to the overall picture, while low values show that participants did not experience difficulties making the ratings. For both bilingual groups, the adverb *evidently* was one of the outliers. For our monolinguals, SPPs for the outliers were as follows: *definitely* = 23%, *apparently* = 21%, followed by *reportedly* = 17%, and *certainly* = 16%.
